# Switching hemophilia A patients to rVIII-SingleChain: The Iberian experience

**DOI:** 10.1097/MD.0000000000039255

**Published:** 2024-09-06

**Authors:** José Manuel Calvo-Villas, Ramiro Núñez-Vázquez, Olga Benítez-Hidalgo, Covadonga García-Díaz, Bernat Galmés, Manuela Carvalho, Pilar Serrano-Torres, José Aznar-Salatti, María Teresa Álvarez-Román

**Affiliations:** a Hematology Department, University Hospital Miguel Servet, Zaragoza, Spain; b Hemophilia Unit, University Hospital Virgen del Rocío, Sevilla, Spain; c Hematology Department, University Hospital Vall d’Hebron, Barcelona, Spain; d Hematology Department, University Hospital of Burgos, Spain; e Hematology Department, University Hospital Son Espases, Palma de Mallorca, Spain; f Congenital Coagulopathies Reference Centre, São João University Hospital Centre, Porto, Portugal; g Commonwealth Serum Laboratories (CSL) Behring, Barcelona, Spain; h Department of Hematology, La Paz University Hospital, Madrid, Spain.

**Keywords:** factor VIII, half-life, hemophilia A, treatment switching

## Abstract

The real-world outcomes of lonoctocog alfa (rVIII-SingleChain), a long-acting factor VIII (FVIII) with a favorable safety and efficacy profile in trials, were assessed in patients with hemophilia A in Iberian (Spain and Portugal). This was a retrospective study involving patients switching to rVIII-SingleChain from other FVIIIs in 7 Spanish and Portuguese hospitals. The efficacy and safety of replacement therapies were compared between 12 months before switching and the period from switching to the end of the study. Twenty-nine patients (median age 25 years; severe hemophilia A, 37.9%) were recruited. Before switching, 12 were on prophylaxis and were followed-up for a median of 12 months. After switching, 17 received prophylaxis with rVIII-SingleChain and were followed-up for a median of 41 months. Those with ≤2 weekly infusions increased from 37.5% before switching to 60.7% after switching to rVIII-SingleChain. The median monthly consumption was 312 IU/kg with prior FVIIIs and 273 IU/kg with rVII-SingleChain. Six spontaneous bleeds were reported in each period in the prophylaxis patients. In the entire cohort, 50 bleeds were reported with prior FVIIIs and 33 were reported after switching to rVIII-SingleChain. Patients requiring ≤1 dose for hemostasis increased from 44.0% with prior FVIIIs to 60.6% with rVIII-SingleChain. Responses were rated good/excellent in 95.4% of cases. No safety concerns were reported. Patients who switched to rVIII-SingleChain prophylaxis had excellent bleeding control and reduced infusion frequency in regular clinical practice, with the subsequent increase in quality-of-life.

## 1. Introduction

Hemophilia A (HA) is a congenital X-linked bleeding disorder that occurs in approximately 25 of every 100,000 live male births^[1]^ and is caused by factor VIII (FVIII) deficiency. It is classified as mild, moderate, or severe according to residual FVIII levels of >5%–<40%, 1%–5%, and <1% of normal, respectively.^[2]^ Manifestations may include spontaneous or traumatic recurrent joint bleeding, damage to the muscles and joints, and excessive internal bleeding that may occasionally be life-threatening. Indeed, HA notably impacts the quality-of-life (QoL), especially in patients with a severe phenotype.^[3]^ Although new therapies for people with HA such as FVIII gene therapy or nonfactor-based therapies have recently emerged,^[4]^ intravenous infusion of FVIII products remain the cornerstone treatment, either on demand according to specific needs or in regular prophylaxis regimens. The World Federation of Hemophilia strongly recommends that patients with a severe hemophilia phenotype have to be on prophylaxis sufficient to prevent articular and musculoskeletal bleeds.^[5]^ The plasma half-life of standard-acting (SHL) FVIII products is in the range of 8 to 12 hours, being approximately 12 hours on average. Thus, patients treated with prophylaxis, especially those diagnosed with severe HA, require frequent intravenous infusions, often ≥3 times a week. Despite the prevention of arthropathy, the burden of prophylaxis may be a major obstacle to adherence. This problem becomes greater in small children and in patients with poor venous access.^[6]^

To address these limitations, long-acting recombinant FVIII (rFVIII) products have been developed with improved pharmacokinetic properties that allow them to show extended half-lives (EHL) in vivo, while retaining efficacy.^[7]^ These long-acting rFVIII products may increase adherence, empower patients, and conserve health resources. Lonoctocog alfa (rVIII-SingleChain [Afstyla^®^]; CSL Behring, King of Prussia, PA) is the first unique truncated B-domain rFVIII with a specific single-chain design obtained by covalently linking heavy and light chains. Single-chain designs increase molecular stability and high binding affinity for von Willebrand factor and, as a result, give a mean half-life in the range of 14 to 15 hours.^[8,9]^

The AFFINITY clinical trial program (phase I/III: NCT01486927; phase III: NCT02093897; phase III extension: NCT02172950) has demonstrated that rVIII-SingleChain not only exhibits an excellent safety profile, but also has excellent hemostatic efficacy for both prevention and treatment of bleeds at all ages, even when dosed 2 to 3 instead of 3 to 4 times a week.^[10–12]^ A detailed review of these studies revealed that prophylaxis with rVIII-SingleChain achieves annualized bleeding rates (ABRs) and percentages of patients with zero bleeds in line with other EHL FVIII preparations, thus substantiating its long-acting nature.^[13]^ The real-world evidence available thus far confirms the clinical trial findings.^[14–19]^ A recent compilation of clinical data encompassing the experience of 48 hospitals in several countries allowed specialists to conclude that the efficacy, dosing pattern, and monthly consumption of rVIII-SingleChain were comparable to those of other EHL products and superior to those of SHL preparations.^[20]^ The current observational study summarizes the experience with rVIII-SingleChain collected in a representative sample of Iberian (Spanish and Portuguese) hospitals on patients with HA and compares it with previous treatments. Dosing frequency and factor consumption in prophylaxis regimens and the dose required to achieve hemostasis when a bleeding event occurred, were assessed in a study cohort before and after the rVIII-SingleChain switch.

## 2. Methods

### 2.1. Study design

This is a multicenter, retrospective observational study, which involved 6 hospitals in Spain (Hospital Universitario La Paz, Madrid; Hospital Universitario Virgen del Rocío, Sevilla; Hospital Universitari Vall d’Hebron, Barcelona; Hospital Universitario Miguel Servet, Zaragoza; Hospital Universitario de Burgos, Burgos; Hospital Universitari Son Espases, Palma de Mallorca) and 1 in Portugal (Centro Hospitalar Universitário de São João, Porto), which manage pediatric or adult HA subjects.

**Figure 1.**
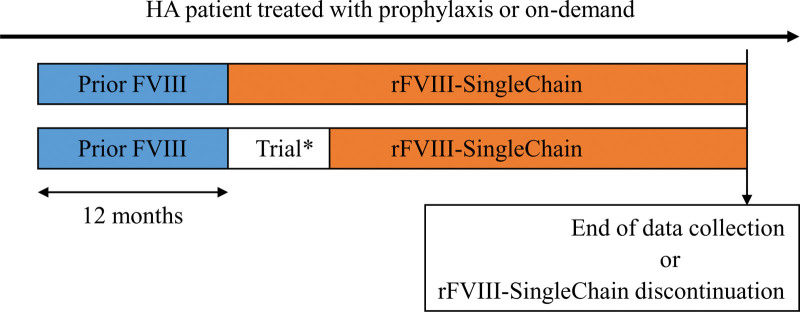
Flowchart diagram of the study. *In the event that patients were enrolled in the Affinity clinical trial program, their data in the trial were not included in the current study. FVIII = factor VIII, HA = hemophilia A, rVIII-SingleChain = single-chain recombinant factor VIII.

The study involved the retrospective collection of preexisting sociodemographic and clinical data from medical records of patients attending the participating hospitals who switched to rVIII-SingleChain for HA treatment. Only data recorded under routine clinical practice were collected for this project (i.e., clinical trial-related data were not considered). Data were collected retrospectively from December 1, 2021 to January 26, 2022. As shown in the flowchart diagram (Fig. 1), the following data were collected: on the one hand, those corresponding to 12 months prior to switching to rVIII-SingleChain; on the other hand, those corresponding to rVIII-SingleChain treatment, from switching to this treatment up to either end of data collection or rVIII-SingleChain treatment discontinuation. In the event that switching to rVIII-SingleChain was due to enrollment in the Affinity program, patients’ data were not considered until they withdrew from the clinical trial.

### 2.2. Participants

Data were collected by the investigators for all cases that met the eligibility criteria in the recruitment period, with no exceptions to avoid risk of bias. The eligibility criteria were as follows: adult, adolescent, or pediatric subjects with a diagnosis of HA who were treated with rVIII-SingleChain for at least 8 consecutive weeks since the start of treatment, regardless of whether this occurred when patients were enrolled in the Affinity program, and who had been treated with another FVIII product for at least 12 months before switching to rVIII-SingleChain.

### 2.3. Collected information

Sociodemographic data of the patients corresponding to the time of switching were collected. Clinical data regarding dosing of FVIII products, number, cause and location of bleeds, management of bleeding events and surgeries, adverse event occurrence, and lifestyle habits, were reported for both periods of the study. The ABRs for all (ABR), spontaneous, traumatic, or joint bleeds, were calculated for the pre- and post-switching periods. The duration of follow-up was reported for each patient. Researcher assessment of hemostatic efficacy was assessed according to the World Federation of Hemophilia guidelines (Supplementary Table 1, Supplemental Digital Content, http://links.lww.com/MD/N355).

### 2.4. Statistical analysis

Continuous variables were reported as median (interquartile range [IQR]) or mean (standard deviation [SD]). The Mann–Whitney *U* test was used to compare the follow-up length, monthly factor consumptions, and ABRs between the pre- and post-switching periods. Chi-square test was used to compare patients with ≥3 versus <3 weekly administrations. Fisher exact test was used to compare the response to treatment (excellent vs other) according to the physician’s perception. When applicable, the numbers of missing data were indicated for each calculation.

### 2.5. Ethics

The study was conducted with the approval of the Independent Ethics Committees of Spain (Hospital Universitario La Paz as the Coordinating Center) and Portugal (Centro Hospitalar Universitário de São João). The ethics committees of the other participating hospitals accepted and applied the requirements of the aforementioned committees. Written informed consent was obtained prior to data collection from each subject, or the subject’s legally accepted representative. The study was conducted in accordance with the Declaration of Helsinki and local regulatory requirements.

## 3. Results

### 3.1. Cohort of study patients

The data were collected between December 2021 and January 2022. Twenty-nine patients meeting all eligibility criteria were recruited from 6 Spanish hospitals and 1 Portuguese hospital (Table 1). Fifteen (51.7%) patients were treated on demand, 12 (41.4%) with prophylaxis, and 2 (6.9%) were treated with both prophylaxis and on demand during the pre-rVIII-SingleChain switch period. After changing to a long-acting rVIII-Single Chain, the proportion of patients receiving prophylaxis was higher than before (58.6%). During the follow-up period of the first part of the study, according to the design which included the last 12 months before switching for all patients, the follow-up period when these were on rVIII-SingleChain treatment lasted a median (IQR) of 35 (15–42) months. The mean of rVIII-SingleChain treatment duration in prophylaxis was 20 months, with a minimum and a maximum of 2 and 38 months, respectively. Four patients were enrolled in the Affinity program immediately after switching to rVIII-SingleChain and remained in the clinical trial until its end. During this period, the data were not considered in the present study. Finally, none of the patients discontinued rVIII-SingleChain treatment during the data collection period of this observational study.

**Table 1:** Cohort characteristics and demographics.

	Whole cohort of patients switching to rVIII-SingleChain (n = 29)
Age at switching (yr), median (IQR)	25.3 (11.2–45.3)
Age group, n (%)	
Pediatric (<12 y.o.)	7 (24.1)
Adolescent (12–17 y.o.)	4 (13.8)
Adult (≥18 y.o.)	18 (62.1)
Ethnicity, n (%)	
Caucasian	28 (96.6)
Afro-American	1 (3.4)
Hemophilia severity at diagnosis, n (%)	
Mild	9 (31.0)
Moderate	9 (31.0)
Severe	11 (37.9)
Product before switching, n (%)*	
Octocog alfa	19 (65.5)
pdFVIII	1 (3.4)
pdFVIII/vWF	2 (6.9)
Moroctocog alfa	6 (20.7)
Turoctocog alfa	1 (3.4)
EHL FVIII	
Rurioctocog alfa pegol	1 (3.4)
Efmoroctocog alfa	1 (3.4)
Treatment before switching, n (%)	
Prophylaxis	12 (41.4)
On-demand	15 (51.7)
Combined	2 (6.9)
Treatment after switching, n (%)	
Prophylaxis	17 (58.6)
On-demand	11 (37.9)
Combined	1 (3.4)

EHL FVIII = extended half-life factor VIII products, IQR = interquartile range, pdFVIII = plasma-derived factor VIII, rVIII-SingleChain = single-chain recombinant factor VIII, SD = standard deviation, vWF = plasma-derived von Willebrand factor, y.o. = years old.

* In the period of 12 mo previous to switching to lonoctocog alfa, 1 patient used moroctocog alfa and turoctocog alfa, and another one, who had 1 traumatic bleed reported during this period, used 2 EHL products, rurioctocog alfa pegol, and efmoroctocog alfa, the first in prophylaxis regimen during 11 mo and 3 wk, and the second on demand in the last week only.

### 3.2. Reasons for switching

Switching to rVIII-SingleChain was decided in order to improve protection in more than half of the cases (55.2%). Achieving better management of bleeding events (20.7%) and reducing infusion frequency or factor consumption (13.8% each) were also other aims that physicians were pursuing by starting rVIII-SingleChain treatment. Discontinuation of prior FVIII commercialization was also the cause of 13.8% of the cases.

### 3.3. Dosing before and after switching in patients treated with prophylaxis

**Figure 2.**
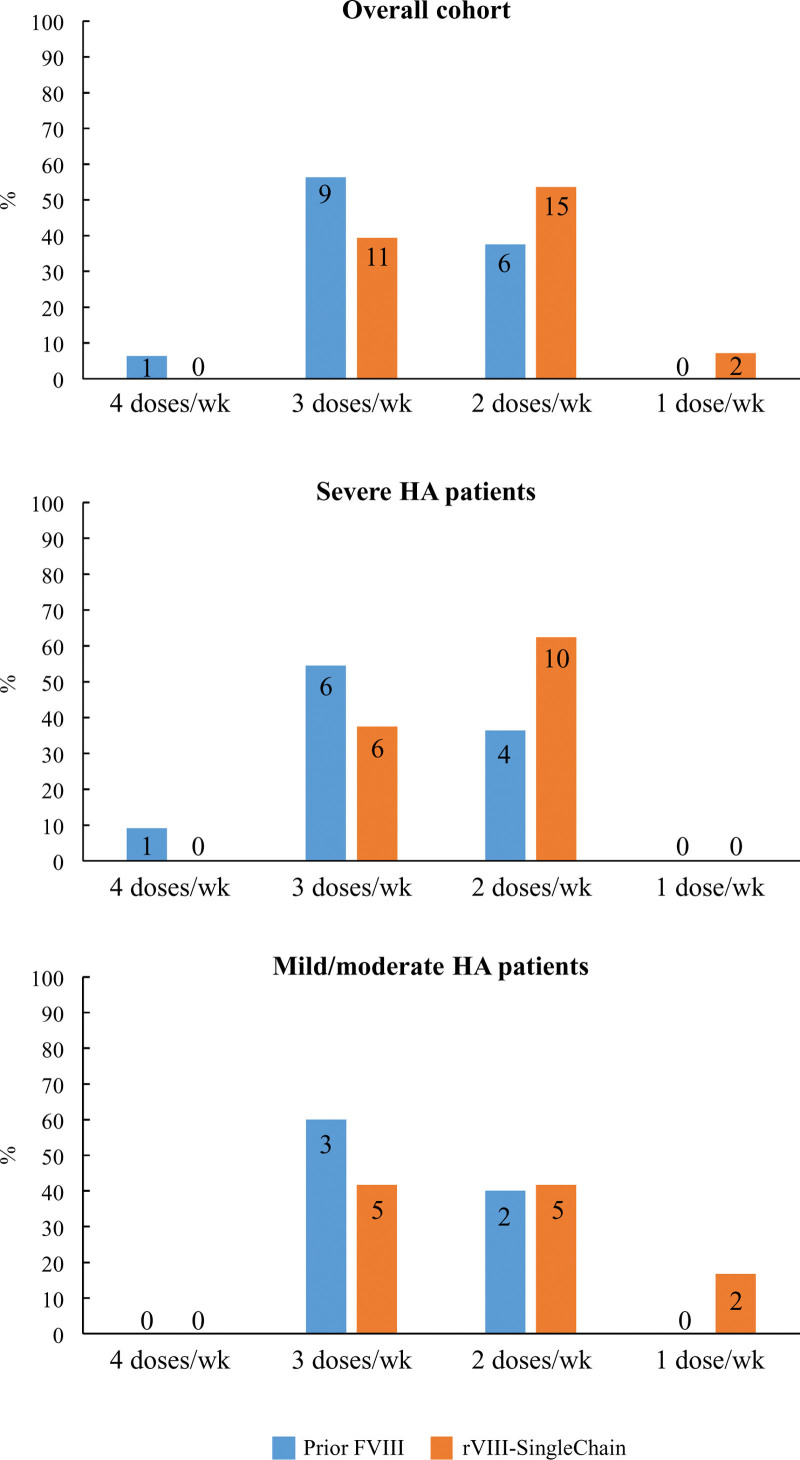
Prophylaxis dosing before and after switching to rVIII-SingleChain. The number of patients treated with prophylaxis was: 14 and 18 before and after switching to rVIII-SingleChain, respectively, in the entire cohort; 10 and 11 before and after switching in the severe HA group; and 4 and 7 before and after switching in the mild/moderate HA group. Histograms represent the percentage of prophylaxis regimens consisting of 1, 2, 3, or 4 weekly doses before (blue) or after (orange) switching, for the overall cohort (top), severe HA patients (middle), and mild/moderate HA patients (bottoms). Several patients were subjected to more than 1 prophylaxis regimen during 1 or another period. Numbers within histograms represent the total number of treatments reported for each condition. HA = hemophilia A.

Considering the overall cohort, 16 and 28 prophylaxis regimens were reported in the pre- and post-switch periods, respectively. The proportion of ≤2 weekly infusion regimens increased in the second period (37.5% vs 60.7% before and after switching, respectively). Accordingly, the number of procedures consisting of ≥3 weekly administrations was reduced (56.3% vs 39.3% before and after switching, respectively, *P* = .069) (Fig. 2, top panel). Patterns consisting of ≤2 weekly infusions were reported in 36.4% and 62.5% regimens among patients with severe HA before and after switching, respectively (*P* = .091) (Fig. 2, middle panel). When the analysis was restricted to mild/moderate HA patients, the proportion of ≤2 weekly infusion patterns increased from 40% to 58.4% after switching to rVIII-SingleChain (*P* = .245) (Fig. 2, bottom panel). Finally, when only the last treatment was considered, a trend toward prophylaxis consisting of fewer infusions was also noted, regardless of severity, when rVIII-SingleChain was used (*P* = .077) (Supplementary Figure 1, Supplemental Digital Content, http://links.lww.com/MD/N354). When patients were categorized according to age, in patients >18 years old, during the pre-rVIII-SingleChain period, 50% of prophylaxis treatments consisted of ≤2 weekly doses. After switching to rVIII-SingleChain, this proportion increased to 71.4% (*P* = .179). The number of reported treatments for the other age groups was too low to draw reliable conclusions.

When considering all patients on prophylaxis, the median (IQR) monthly factor consumption was 312 (230–498) IU/kg/mo and 273 (187–490) IU/kg/mo for prior FVIII and rFVIII-SingleChain, respectively, *P* = .265. There were no remarkable differences when the analysis was limited to severity or age groups. The mean infusion dose, normally in the range of 30 to 35 IU/kg, did not differ notably between the pre- and post-switching periods, either in the entire cohort or when patients were categorized according to severity or age.

### 3.4. Bleeds during the study

The number of bleeds and ABRs in both pre- and post-switching periods are summarized in Table 2 for the entire cohort and for those patients who were treated with either prophylaxis or on-demand. When using their prior FVIII products for prophylaxis, 10 of 12 patients (83.3%) had zero total bleeds in 12 mo, and the 2 remaining patients had 5 and 1 spontaneous bleeds, respectively, in the same period. Eight (47.1%) and 12 (70.6%) out of 17 patients had zero total and spontaneous bleeding, respectively, while on prophylaxis with rVIII-SingleChain treatment for 41 (24–62) months.

**Table 2:** Bleeds and annualized bleeding rates in the periods before and after switching to rVIII-SingleChain.

	Whole cohort		*P*	Prophylaxis*			On demand*		
	Prior FVIII (n = 29)	rVIII-SingleChain (n = 29)		Prior FVIII (n = 12)	rVIII-SingleChain (n = 17)	*P*	Prior FVIII (n = 15)	rVIII-SingleChain (n = 11)	*P*
Follow-up (mo), median (IQR)	12 (12–12)	35 (15–42)	–	12 (12–12)	41 (24–62)	–	12 (12–12)	23 (11–36)	–
Bleeds, n									
Total	50	33	–	9	15	–	17	17	–
Spontaneous	34†	10	–	6	6	–	6	4	–
Traumatic	16	23	–	3	9	–	11	13	–
Hemarthrosis	37	17	–	5	9	–	9	8	–
ABR									
Median (IQR)	0 (0–1.5)	0.3 (0–0.6)	.121	0 (0–0)	0.1 (0–0.5)	.163	1.0 (0–2.0)	0.3 (0–2.2)	.763
Mean (SD)	1.7 (4.4)	0.6 (1.0)		0.7 (1.8)	0.3 (0.4)		1.1 (1.5)	0.9 (1.4)	
AsBR									
Median (IQR)	0 (0–1.0)	0 (0–0)	.046	0 (0–0)	0 (0–0.2)	.658	0 (0–1.0)	0 (0–0)	.204
Mean (SD)	1.2 (4.1)	0.1 (0.3)		0.5 (1.4)	0.1 (0.2)		0.4 (0.6)	0.1 (0.4)	
AtBR									
Median (IQR)	0 (0–1.0)	0 (0–0.3)	.368	0 (0–0)	0 (0–0.3)	.247	0 (0–1.0)	0.3 (0–1.3)	.488
Mean (SD)	0.5 (1.1)	0.4 (0.9)		0.2 (0.9)	0.2 (0.4)		0.7 (1.4)	0.8 (1.3)	
AjBR									
Median (IQR)	0 (0–1.0)	0 (0–0.3)	.242	0 (0–0)	0 (0–0.2)	.157	0 (0–1.0)	0 (0–0.7)	.789
Mean (SD)	1.3 (4.1)	0.3 (0.8)		0.4 (1.4)	0.2 (0.4)		0.6 (0.9)	0.6 (1.2)	

In the period after switching to rVIII-SingleChain, the ABRs were calculated for each patient as the number of bleeds during the time in the treatment period of the study in days, divided by 365.

ABR = annualized bleeding rate, AjBR = annualized joint bleeding rate, AsBR = annualized spontaneous bleeding rate, AtBR = annualized traumatic bleeding rate, FVIII = factor VIII, IQR = interquartile range, rVIII-SingleChain = single-chain recombinant factor VIII, SD = standard deviation.

* Those patients who combined prophylaxis and on-demand regimens in the same period, either pre-switching period or rVIII-SingleChain period, are not considered.

† One patient who was treated on-demand in the first 11 mo and then changed to prophylaxis, had 22 spontaneous bleeds in the 12-mo period before switching to rVIII-SingleChain.

Thirty-four bleeds, of which 10 were spontaneous and 24 were traumatic, were reported in patients who were treated on demand. Half of the events occurred before switching and the other half occurred with rVIII-SingleChain. Finally, when considering the entire cohort of participants (n = 29), the value of ABRs was zero in 13 of 16 cases, and the annualized spontaneous bleeding rate was lower after switching to rVIII-SingleChain. No significant differences were observed between the 2 study periods.

### 3.5. Management of bleeding events

**Figure 3.**
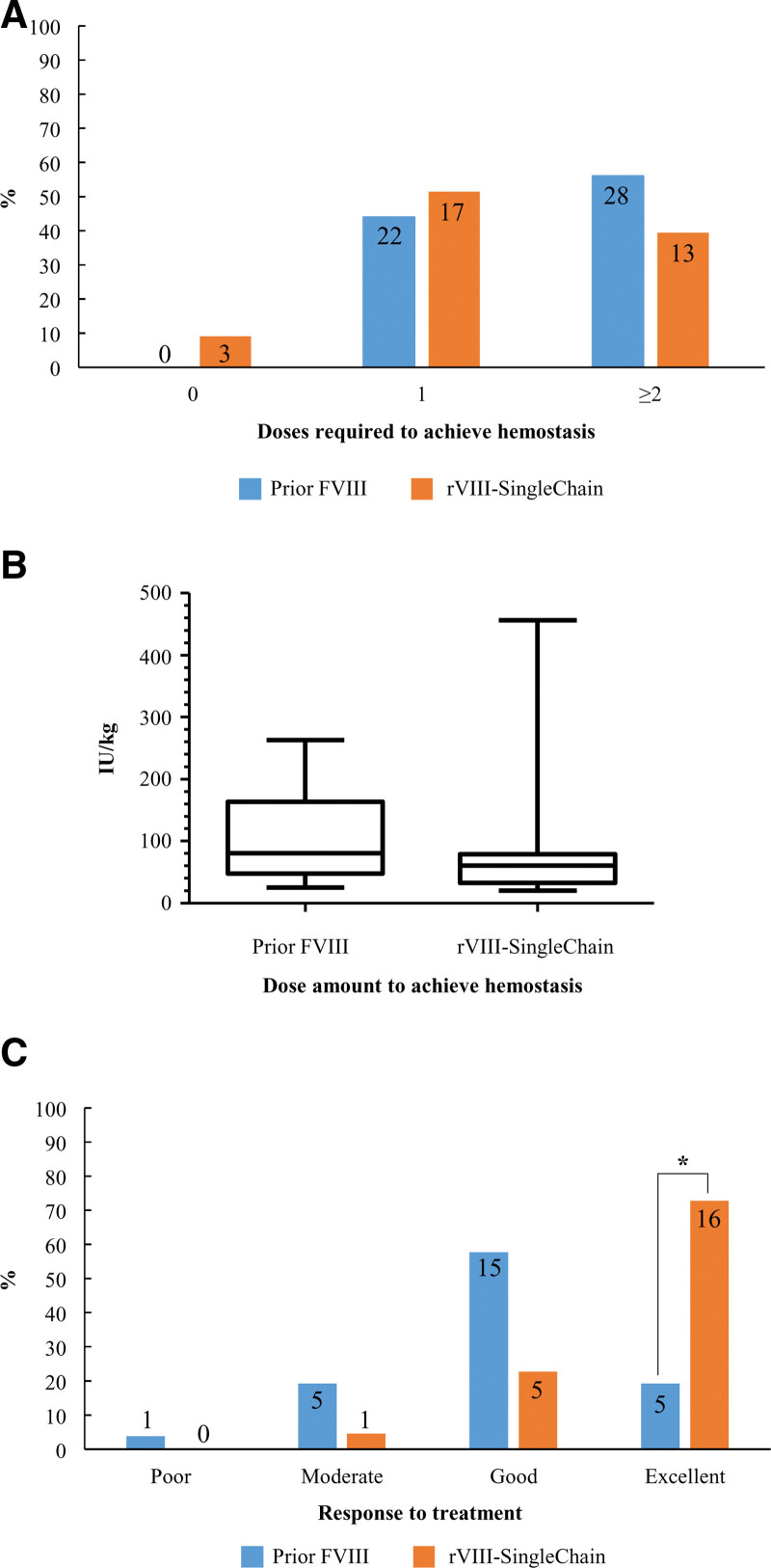
Dose and efficacy of prior FVIII and rVIII-SingleChain to treat bleeding events. The number of doses required to achieve hemostasis (A), the median (IQR) total dose used to achieve hemostasis (B), and hemostasis efficacy as rated by the researcher (C) are shown for treatment of bleeding events with prior FVIII and rVIII-SingleChain. In panels (A and C), the numbers within the histograms represent the number of events reported under the corresponding condition. Data regarding total dose to achieve hemostasis were not available for 29 out of 50 bleedings and 9 out of 33 bleedings in the pre- and post-switching periods, respectively. Data regarding the response to treatment were not available for 24 and 8 bleedings before and after switching, respectively. *P < .001 according to Fisher exact test (excellent vs others). FVIII = factor VIII, IQR = interquartile range, rVIII-SingleChain = single-chain recombinant factor VIII.

Eighty-three bleeding events were reported in the entire cohort (n = 29) during both study periods, 50 and 33 of which occurred before and after switching, respectively. All of them were successfully resolved, that is bleeding stopped upon hemostatic treatment. Figure 3 summarizes the most relevant results for this area. Forty-four percent of bleeds treated with the prior FVIII product required ≤1 dose to achieve hemostasis, whereas this occurred in 60.6% of those treated with rVIII-SingleChain (Fig. 3A). The median total dose required for bleeding cessation was on average 20 IU/kg higher when prior FVIII was used, although variability among episode requirements was high (80 [42–159] vs 60 [36–82] IU/kg) (Fig. 3B). Finally, according to Fisher exact test, the number of bleeding events whose response to treatment was rated as excellent by researchers was significantly higher when rVIII-SingleChain was used instead of prior FVIII (16 of 22 vs 5 of 26, *P* < .001) (Fig. 3C).

One invasive dental procedure was undergone by 1 patient before switching. Hemostasis was not achieved with the FVIII product. No additional surgeries were performed during that period. Seven surgical procedures, including 1 ankle synoviorthesis, were documented during the follow-up period after switching to rVIII-SingleChain (Supplementary Table 2, Supplemental Digital Content, http://links.lww.com/MD/N355). One infusion was sufficient to achieve hemostasis in 4 of these cases, the median (IQR) total dose to reach this goal was 50.0 (27.5–80.0) IU/kg, and response to treatment was rated as good or excellent in 6 out of 7 procedures.

### 3.6. Lifestyle before and after switching

The use of rVIII-SingleChain involves changes in the lifestyle of the patients. Physicians perceived an increase in physical activity intensity after switching (Supplementary Figure 2, Supplemental Digital Content, http://links.lww.com/MD/N354).

### 3.7. Safety

Two patients (6.9%) had a history of use of FVIII inhibitors. There was no inhibitor occurrence in the 12 months with prior FVIII, and inhibitors were not reported either after switching to rVIII-SingleChain or during the remainder of the follow-up period. Neither thromboembolic events nor other adverse events related to FVIII-based treatments were observed throughout the study periods.

## 4. Discussion

This is the first report documenting the experience of rVIII-SingleChain in Iberia. The results are consistent with those reported previously. In our cohort, all but one of those patients with prophylaxis used SHL products in the first phase of the study. While 62.5% of the treatments administered during this period consisted of ≥3 weekly doses, only 39.3% of patients on prophylaxis after switching had ≥3 weekly doses. Thus, the proportion of treatments consisting of 2 or fewer doses was >60% in the second phase of the study. This trend toward lower dosage frequencies with rVIII-SingleChain treatment was independent of severity. These findings are in line with those reported by previous real-world studies, where the proportion of patients administered <3 weekly doses of rVIII-SingleChain ranged from 60% to 75%.^[19,20]^ The structural hallmarks of rVIII-SingleChain confer a favorable pharmacokinetic profile compared to SHL products. The trough levels of rVIII-SingleChain at 48 hours after dosing were shown to be nearly 4-fold higher than those measured after a SHL product was administered, and doses of 50 IU/kg succeeded in maintaining circulating FVIII activity levels above 1% for more than 5 days, similar to other EHL preparations.^[21]^ Thus, the circulating half-life of rVIII-SingleChain allows dosing at less frequent intervals than SHL products, while maintaining a good efficacy and safety profile.^[9]^ A recent study confirmed that the PK-related advantages associated with the use of rVIII-SingleChain can also be observed in real-world patient series.^[19]^

The lower dosage frequency of rVIII-SingleChain was accompanied by encouraging results regarding its efficacy in preventing bleeding. Considering prophylaxis treatments only, the aim of zero spontaneous bleeding was achieved by more than 70% of patients after switching to rVIII-SingleChain, a proportion that was comparable to that of patients with zero spontaneous bleeds with their prior FVIII namely around 80%. Since the median follow-up period was more than 3-fold greater when prophylaxis was administered with rVIII-SingleChain, this result is noteworthy. Our findings regarding protection against bleeding are also in line with those described in the literature. Sixty percent of 129 patients who were treated with rVIII-SingleChain in centers in Germany, Italy, and the United States reported zero bleeds during a mean follow-up period of 47 months, a percentage that was similar to that observed in 159 patients treated with an EHL product, namely rFVIII Fc fusion protein, and higher than that observed in 328 patients treated with 2 SHL products, octocog alfa and BAY 81-8973.^[20]^ The results of the 1-country studies encompassed by this pooled analysis, and others did not differ remarkably from these.^[14–17,19]^ Furthermore, bleeding prevention has been shown to be superior to rVIII-SingleChain than with other EHL FVIII products at comparable dosage frequencies in several cohorts.^[14,16]^

The monthly consumption of replacement therapy product in patients on prophylaxis slightly decreased when rVIII-SingleChain was used instead of the prior FVIIIs. In the entire cohort, the monthly median value was 39 IU/kg (8%) lower in the post-switching period than that reported in the first phase. The difference was exactly the same when only those patients diagnosed with severe HA were considered, and was 97 IU/kg lower in the moderate HA group. These comparisons did not reach statistical significance, which could be partly due to the wide intragroup variability as well as to sample size limitation. In daily practice, rVIII-SC dose is decided according to clinical criteria to warrant hemostatic protection beyond cost-related considerations. In our study, the median weekly consumption of rVIII-SingleChain was 63 IU/kg, slightly lower than the 86 IU/kg reported for the pooled multicountry analysis of real-world series, which was 15 IU/kg lower than that reported for octocog alfa in the same cohorts.^[20]^ The available real-world studies confirm that the consumption of rVIII-SingleChain is more than 10% lower than that reported for SHL products.^[14–16,18,19]^ Thus, the use of rVIII-SingleChain may contribute to saving FVIII product while maintaining or improving the level of bleeding protection conferred by the SHL preparations.

A shift toward a lower dosage requirement to achieve blood cessation when bleeding events occurred was perceived after switching to rVIII-SingleChain. At least 2 doses were needed in 56.0% of the 50 bleeds occurring when patients were being treated with their prior FVIII products, whereas this occurred in only 39.4% of the 33 bleeds managed with rVIII-SingleChain. Thus, 60.6% of bleeds treated with rVIII-SingleChain did not require a second dose. Unsurprisingly, the median amount of product required to resolve hemorrhage was lower when rVIII-SingleChain was used. Physicians rated as excellent the response to treatment in 16 out of 22 bleeds managed with rVIII-SingleChain, while this had occurred in only 5 out of 26 bleeds treated with prior FVIII products. The improvement perceived when rVIII-SingleChain was used to manage bleeding events, irrespective of whether patients were treated with prophylaxis or on-demand, has also been assessed in other real-world settings.^[14–16,22]^ On the other hand, rVIII-SingleChain was effective to manage bleeding in the 7 surgical procedures that the patients in our study underwent after switching. Physicians rated the response as good or excellent to rVIII-SingleChain in all but 1 procedure, in line with the previously described outcomes.^[10,12,23–25]^

Neither inhibitors nor thromboembolic events were reported during the follow-up period of patients on prophylaxis with rVIII-SingleChain, thus confirming the safety profile of this therapy that had been previously noted in clinical trials and post-marketing surveillance.^[12,20]^ Finally, it is worth mentioning that physicians perceived an increase in the level of physical activity of patients after switching to the rVIII-SingleChain. The reduction in dosing frequency accompanied by an adequate level of protection against spontaneous bleeding may have contributed to reinforcing patients’ confidence in therapy and, thus, improve their QoL.^[26]^ Importantly, among those FVIII products funded by Spanish and Portuguese healthcare services, rVIII-SingleChain has the lowest cost per IU, with the subsequent benefit in economic terms for Spanish/Portuguese administrations while keeping ABRs at appropriate levels. The reduction in dosing frequency may also explain the finding that the proportion of patients who chose prophylaxis after changing to rVIII-SingleChain, increased to nearly 60%. Therefore, an improvement in adherence after switching to rVIII-SingeChain can be envisaged. Nevertheless, understanding why patients choose to switch, and what they expect, is important for identifying which patients are likely to benefit most from such a change.^[27]^

Our study has limitations. The multicentric, retrospective nature of the study meant that treatments were decided according to each center’s criteria. As a result, the FVIII products used before switching varied within the cohort. The sample size may not be sufficiently powered to detect subtle differences between groups. The occurrence of a bias associated with the subjective judgment of the different researchers when assessing bleeding or lifestyle habits cannot be entirely ruled out. During the trial period, adherence may have influenced the outcomes positively. Categorizations by regime (prophylaxis vs on-demand before and after switching) and age groups precluded the obtaining of large sample sizes for subgroups. Information regarding patients’ own perception of change in lifestyle habits was not collected. Finally, the influence of ethnicity could not be assessed since only 1 patient was not of Caucasian origin.

## 5. Conclusion

In summary, the Iberian experience with rVIII-SingleChain aligns with that gained from clinical trials and daily clinical practice studies. At a lower dosing frequency than SHL products, rVIII-SingleChain protects HA patients from bleeding regardless of age or disease severity in all clinical settings, namely prophylaxis, on-demand treatment, or surgery. Switching to rVIII-SingleChain may contribute to increase activity habits that can be reflected in an improvement in the QoL of patients with HA, especially those with poor venous access or enjoying an active lifestyle.

## Acknowledgments

The authors would like to thank the patients for their participation in the study and study personnel for their care. The authors also thank the participating centers: Hospital Universitario Virgen del Rocío, Sevilla; Hospital Universitario Miguel Servet, Zaragoza; Hospital Universitario Vall d’Hebron, Barcelona; Hospital Universitario de Burgos; Hospital Universitario Son Espases, Palma de Mallorca, Hospital Universitario La Paz, Madrid, Spain; and São João University Hospital Centre, Porto from Portugal. The authors would also like to thank Paulo Pereira, Vera Vicente, and Andreia Carapinha (CTI Clinical Trial and Consulting Services, Portugal) for data management and statistical support; Dr Ramón Montes (Ambos Marketing Services, Spain) for providing medical writing support and editing; and Lluis Riera, Patricia Martínez, and Joana Rodríguez (CSL Behring) for their insights on the manuscript writing and thorough review.

## Author contributions

**Conceptualization:** Jose Manuel Calvo-Villas, Ramiro Núñez-Vázquez, Pilar Serrano-Torres, José Aznar-Salatti, María Teresa Álvarez-Román.

**Data curation:** Jose Manuel Calvo-Villas, Pilar Serrano-Torres, José Aznar-Salatti, María Teresa Álvarez-Román.

**Investigation:** Jose Manuel Calvo-Villas, Ramiro Núñez-Vázquez, Olga Benítez-Hidalgo, Covadonga García-Díaz, Bernat Galmés, Manuela Carvalho, María Teresa Álvarez-Román.

**Project administration:** Jose Manuel Calvo-Villas, María Teresa Álvarez-Román.

**Supervision:** Jose Manuel Calvo-Villas, Pilar Serrano-Torres, José Aznar-Salatti, María Teresa Álvarez-Román.

**Visualization:** Jose Manuel Calvo-Villas, Pilar Serrano-Torres, José Aznar-Salatti, María Teresa Álvarez-Román.

**Writing—review & editing:** Jose Manuel Calvo-Villas, Pilar Serrano-Torres, María Teresa Álvarez-Román.

**Methodology:** Jose Manuel Calvo-Villas, Ramiro Núñez-Vázquez, Olga Benítez-Hidalgo, Covadonga García-Díaz, Bernat Galmés, Manuela Carvalho, María Teresa Álvarez-Román.

**Formal analysis:** Pilar Serrano-Torres, José Aznar-Salatti, María Teresa Álvarez-Román.

**Funding acquisition:** Pilar Serrano-Torres, José Aznar-Salatti.

**Validation:** Pilar Serrano-Torres, José Aznar-Salatti, María Teresa Álvarez-Román.

**Writing—original draft:** Pilar Serrano-Torres, José Aznar-Salatti, María Teresa Álvarez-Román.

## Supplementary Material


