# Supplementary material for: Switching hemophilia A patients to rVIII-SingleChain: The Iberian experience
Source: Medicine (Baltimore). 2024 Sep 6;103(36):e39255. doi: 10.1097/MD.0000000000039255 (PMC11384051; doi:10.1097/MD.0000000000039255)
Supplement: Supplementary file 1 [file medi-103-e39255-s001.docx]

**Supplementary Table 1. Criteria for researcher assessment of hemostatic efficay according to WFH guidelines**

| **Category** | **Description** |
| --- | --- |
| Excellent | Complete pain relief and/or complete resolution of signs of continuing bleeding after the initial infusion within 8 h and not requiring any further factor replacement therapy within 72 h after onset of bleeding |
| Good | Significant pain relief and/or improvement in signs of bleeding within approximately 8 h after a single infusion but requiring more than 1 dose of factor replacement therapy within 72 h for complete resolution |
| Moderate | Modest pain relief and/or improvement in signs of bleeding within approximately 8 h after the initial infusion and requiring more than 1 infusion within 72 h but without complete resolution |
| Poor | No or minimal improvement, or condition worsens, within approximately 8 h after the initial infusion |

Adapted from Srivastava A et al, Haemophilia 2020;26 Suppl 6:1-158.

WFH, World Federation of Hemophilia.

**Supplementary Table 2. Surgical procedures after switching to rVIII-SingleChain**

| Procedure | Doses | Total amount  rVIII-SingleChain (IU/kg) | Hemostasis | Response |
| --- | --- | --- | --- | --- |
| Urinary bladder polypectomy | 2 | 50.0 | Yes | Moderate |
| Invasive dental procedure | 1 | 50.0 | Yes | Excellent |
| Umbilical hernia repair surgery | 1 | 43.5 | Yes | Excellent |
| Strabismus surgery | 1 | 27.5 | Yes | Excellent |
| Corneal ring implantation for keratoconus | 1 | 20.0 | Yes | Good |
| Arthroscopy | 2 | 80.0 | Yes | Good |
| Ankle synoviortesis* | 5 | 150.0 | Yes | Good |

The total number of injections encompasses those administered pre-surgery and those up to 14 days post-surgery.

*One patient had 1 pre-surgery and 1 post-surgery (24 hr) rVIII-SingleChain dose and, furthermore, 3 additional infusions were required 3, 5 and 7 days after surgery, due to an intense inflammatory reaction.

hr = hours.
