# Supplementary material for: Switching hemophilia A patients to rVIII-SingleChain: The Iberian experience
Source: Medicine (Baltimore). 2024 Sep 6;103(36):e39255. doi: 10.1097/MD.0000000000039255 (PMC11384051; doi:10.1097/MD.0000000000039255)
Supplement: Supplementary file 2 [file medi-103-e39255-s002.docx]

**Supplementary Figure 1. Prophylaxis dosing when only the last treatment received by each patient with either prior FVIII or rVIII-SingleChain was considered**

**
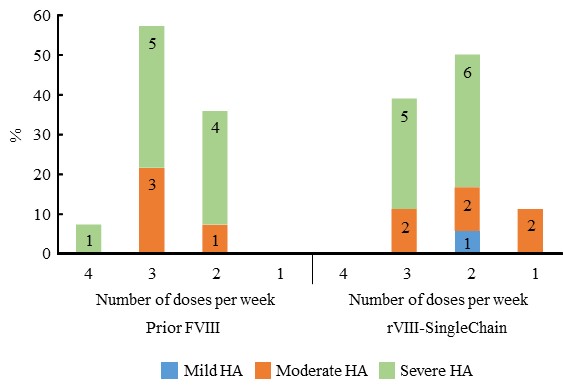
**

Fourteen and 18 patients were treated with prophylaxis in the periods corresponding to the use of prior FVIII or rVIII-SingleChain, respectively. Numbers within histograms are the total number of treatments reported for each condition according to HA severity.

**Supplementary Figure 2. Physical activity level of patients according to physicians’ perception**


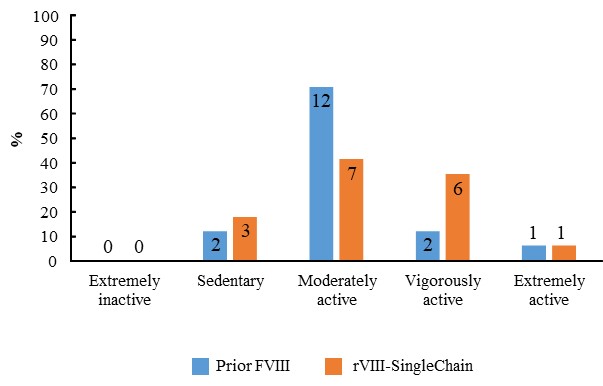


Data regarding the extent of physical acivity are missing for 12 patients.

FVIII = factor VIII, rVIII-SingleChain = single-chain recombinant factor VIII.
